# Integrative diagnostics of the gastro-intestinal tract – gastroesophageal reflux and constipation in practice

**DOI:** 10.1007/s00247-023-05757-9

**Published:** 2023-09-19

**Authors:** Erich Sorantin, Andrea Huber-Zeyringer

**Affiliations:** 1 Division of Pediatric Radiology, Department of Radiology, Medical University Graz, Auenbruggerplatz 34, A – 8036 Graz, Austria; 2 Department of Pediatric and Adolescent Surgery, Medical University Graz, Auenbruggerplatz 34, A – 8036 Graz, Austria

**Keywords:** Constipation, Diagnostic imaging, Esophageal pH monitoring, Fluoroscopy, Gastroesophageal reflux, Manometry

## Abstract

Common disorders of the gastrointestinal (GI) tract, such as gastroesophageal reflux/disease (GER/D) and constipation, are frequent causes for seeking medical support in infants. Diagnostic workup must ensure that diagnosed diseases are responsible for such complaints, thus enabling appropriate therapy. In this context assessment consists of clinical examination, functional tests, and imaging, which should be done in a staged manner. Close cooperation between clinicians and clinical radiologists enables optimal diagnostics, thus forming the basis for appropriate therapy.

Disorders of the gastrointestinal (GI) tract are frequent causes for seeking medical support in infants – among them, gastroesophageal reflux/disease (GER/D) and constipation are common [1, 2]. Diagnostic workup must ensure that diagnosed diseases are responsible for the complaints – thus enabling appropriate therapy.

Several tools are available for GI diagnostic work-up – functional and imaging. These tools should be used in a staged manner in order to ensure highest diagnostic effectiveness, thereby following the ALARA (“As Low As Reasonably Achievable”) principle. Moreover, gastroesophageal reflux (GER) has to be differentiated from gastroesophageal reflux disease (GERD) – in the latter, children suffer from failure to thrive and anemia, and tests for occult blood in stools are positive.

This article provides a comprehensive overview of diagnostic work-up in GER and constipation. For this purpose, the paper will be structured in the following way: clinical and functional assessment, followed by imaging.

## Gastroesophagel reflux: clinical and functional assessment

Gastroesophageal reflux (GER) is a condition found in infants, with symptoms peaking in the fourth month of life. The disease affects about 10% of all infants and resolves in over 90% of cases by age one. In 60% of cases, healing takes 18 months; in 30%, up to the fourth birthday [3].

GER represents a maturation disorder of the lower esophageal sphincter due to the sphincter’s frequent spontaneous relaxations; a reduced lower esophageal sphincter resting pressure is also contributory and is considered to be a sign of higher-grade reflux disease. In addition, failure of the infantile physiological movement disorder of the esophagus to mature and resolve normally within the first year of life is also contributory to GER in infancy.

GER disease (GERD) is an important comorbidity of certain underlying diseases; as such, children with congenital malformations of the esophagus, the diaphragm, and the abdominal wall are considered at risk patients. In children, after corrected esophageal atresia, tension on the esophageal stump causes the His angle to be abolished and consequently GER/D occurs, which poses a challenge in treatment due to the existing peristaltic disturbance of the esophagus.

Children with long-gap esophageal atresia and diaphragmatic hernias are particularly affected – especially those with left-sided hernias because the sphincter apparatus is not outlined on the left-hand side by the diaphragmatic crus. It should be mentioned that symptoms of GER may overlap with those of other gastrointestinal motility disorders, like gastroparesis or chronic intestinal pseudo-obstruction.

Moreover, GER/D is a frequent comorbidity in patients suffering from multiple disabilities and, due to difficulties in direct communication, nutrition is often not given the attention it deserves.

In the event of non-treatment, complications occur in 10% of patients, broken down as follows: 5% will develop an esophagitis/stricture, 5% will die due to recurrent aspiration or malnutrition [4].

Therapy-resistant iron deficiency anemia, severe failure to thrive, recurrent aspiration, and nocturnal restlessness can be symptoms of existing GERD. Unfortunately, GERD is often diagnosed in late disease stages, consequently challenging surgical rehabilitation and postoperative, interdisciplinary management.

GERD symptoms in infants that require investigation are as follows: (a) symptoms related to volume reflux: vomiting, dystrophy/feeding problems, underweight; (b) pulmonary symptoms: micro-aspiration and coughing during the night (the recumbent position leading to increased incidence of reflux), acute life-threatening events, recurrent pneumonia; and (c) symptoms due to acid exposure: pain, iron deficiency (micro-bleeding), esophagitis, and Barrett’s esophagus [5].

The following GER tests are available:

*Impedance-pH-metry* is today’s gold standard for GER assessment. It is a standardized method of gastroenterological functional diagnostics in which the pH value and electrical impedance are continuously measured between several measuring points in the esophagus. Compared to ph-monitoring, it enables the detection of solid, liquid, and gaseous gastroesophageal reflux, regardless of pH value. In addition, reflux height can be determined and antegrade and retrograde bolus transit can be assessed.

**Fig. 1:**
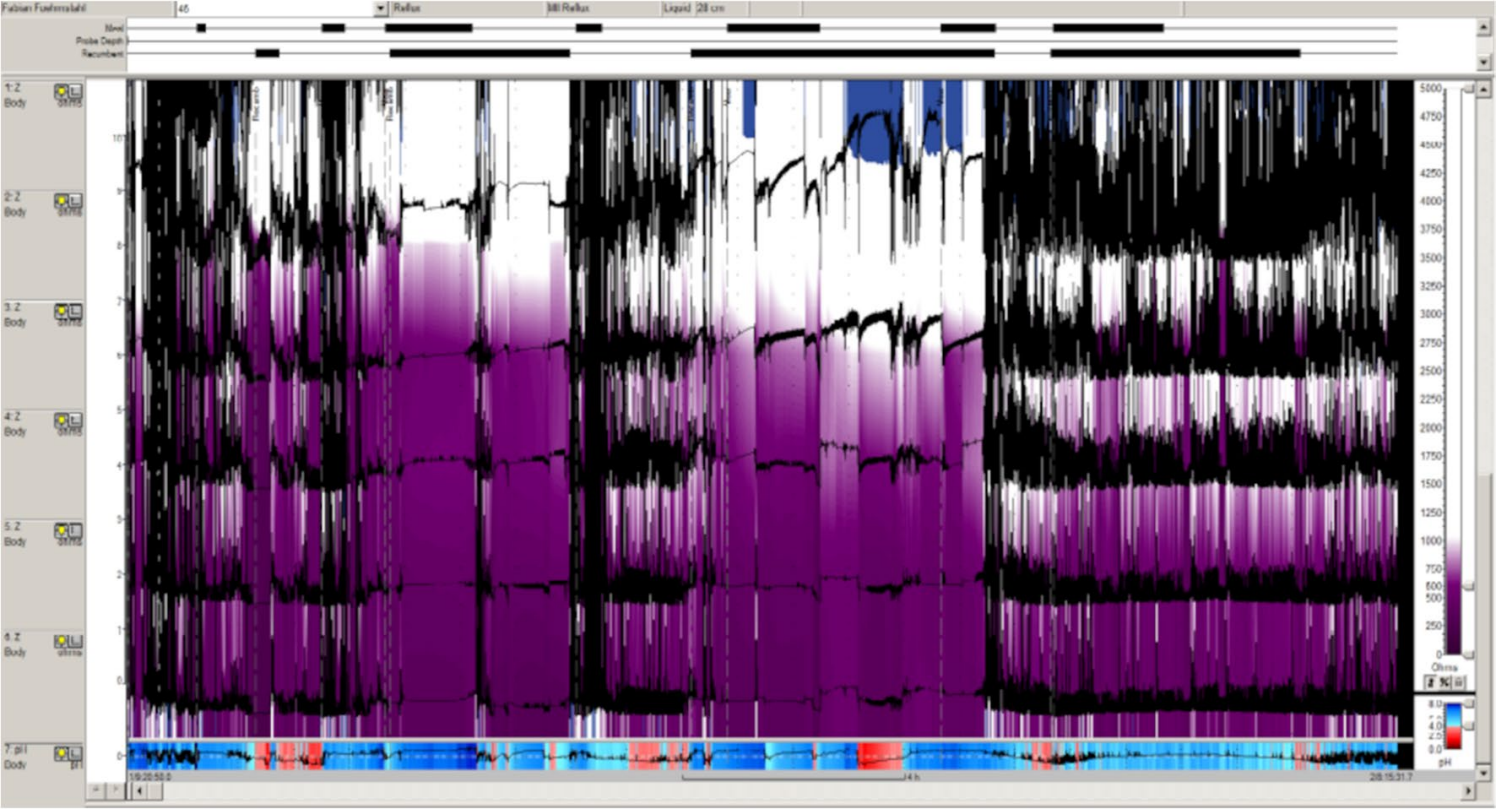
A color-coded screenshot from an impedance-pH-metry in a 14-year-old dystrophic boy with a history of tetraparesis and feeding via a percutaneous endoscopic gastrostomy button. The patient was said to tolerate only 180 ml nutrition, vomiting at volumes above this. The screenshot shows a massive decrease in electrical impedance (*violet*) which is a known marker for severe esophagitis and/or Barrett’s esophagus

The probe is inserted into the esophagus and left in place for at least 22 h. It is an outpatient examination, and the patient’s normal everyday routine should be maintained. Meals, bedtime, and symptom documentation allows for reflux symptom correlation. An example is shown in Fig. 1, and impedance-ph-metry advantages and disadvantages are listed in Table 1.

**Table 1:** Advantages and disadvantages of impedance-pH-metry

Impedance-ph-metry	
Advantages	Disadvantages
Superior to 24-h pH monitoring, radiology, and endoscopy as a single examination for GER diagnosis	Only available in some centers, evaluation requires experience
Determines level of reflux episodes and thus also correlates extraesophageal symptoms (cough, hoarseness, bradycardia)	In pronounced mucosal changes (Barrett’s mucosa/esophagitis), the extent of the reflux is not sufficiently shown
More extensive diagnostics in infants and young children, since most reflux at this age is non-acidic or weakly acidic	No uniform normal limits for infants, children
Differentiates between GER, hypersensitive esophagus, and functional heartburn	
Assesses the bolus transit and thus the function of the esophagus [4]	
Identifies aerophagia and air reflux and can give indications of esophagitis/Barrett’s mucosa	

*GER* gastroesophageal reflux

*Esophageal manometry* is used to derive esophageal pressure tracings using a nasogastric measuring probe. Nowadays, in addition to water-perfused measuring systems, high-resolution electronic measuring catheters are also available.

**Fig. 2:**
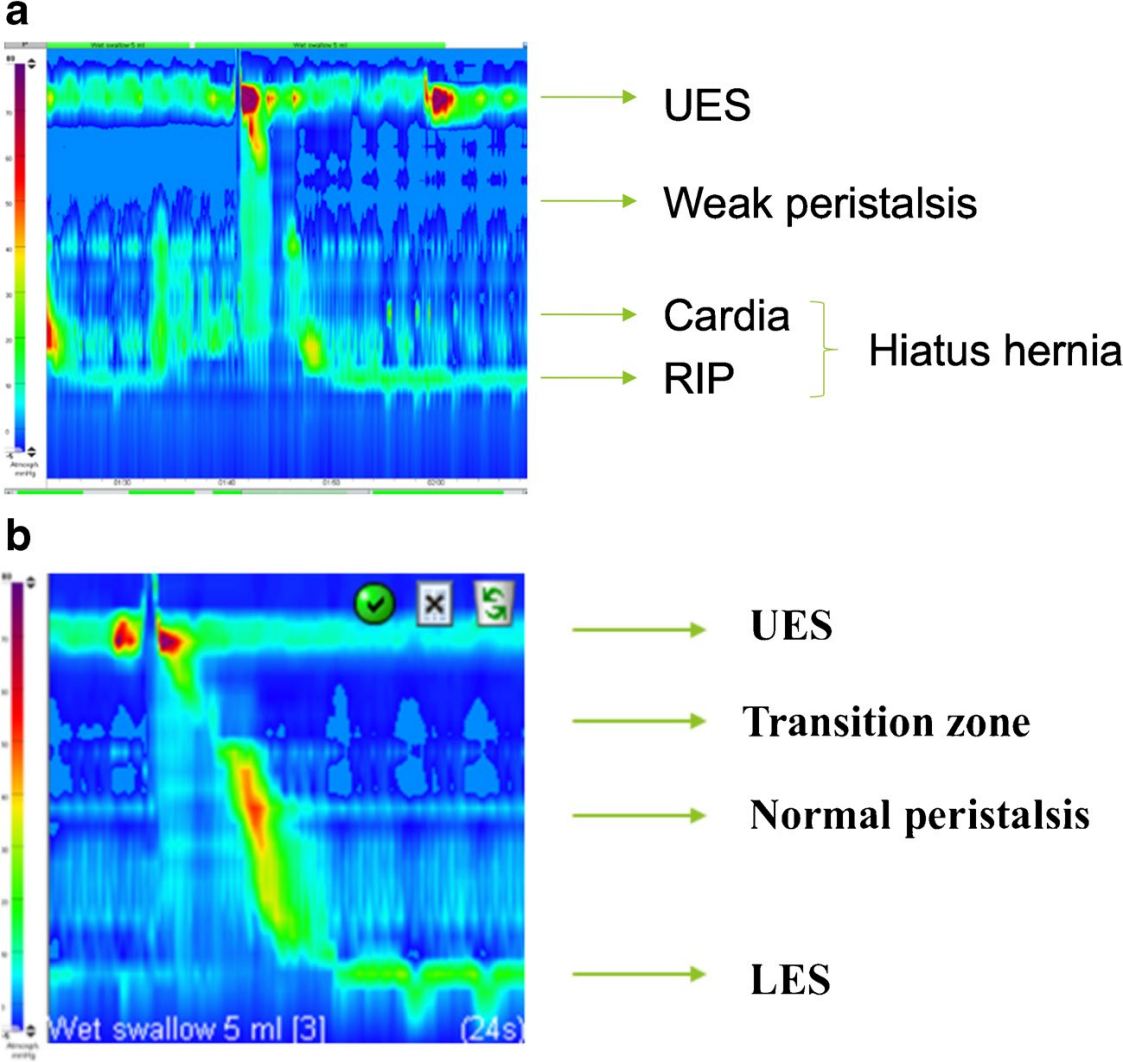
Pressure gradient color-coded screenshots (color bar on the left) from esophageal manometry studies in an 8-year-old girl who, during surgery for partial volvulus in the first year of life, was found to have a hiatus hernia. **a** The preoperative study shows an absence of the normal peristalsis wave which should be running obliquely downwards from left to right. **b** The postoperative study shows a normal peristaltic wave *LES* lower esophageal sphincter, *RIP* respiratory inversion points (represent the level of the diaphragm), *UES* upper esophageal sphincter

High-resolution esophageal manometry enables tracking of the contraction processes of the entire esophagus to be displayed at defined intervals (with the help of many electronic pressure transducers), as well as depiction of the position, contractility, and relaxation of the esophageal sphincters (Fig. 2).

Esophageal manometry is used to clarify dysphagia and chest pain of non-cardiac origin. It is the gold standard for diagnosing motility disorders such as achalasia.

Since the introduction of impedance-pH-metry, high-resolution esophageal manometry does not play a role. The only exception is for the determination of lower esophageal sphincter position as needed for impedance-pH-metry if a chest radiograph is either not possible or not consented for.

*24-h pH monitoring* is a simple, standardized examination method in gastroenterology. It continuously monitors esophageal pH value and enables quantitative assessment of acid GER.

After a 4-h fasting phase, a pH probe is inserted into the esophagus and pH assessment is performed for at least 22 h. This can be performed on an outpatient basis. Careful documentation of daily routine—including meals, bedtimes, and symptoms—allows for GER symptom correlation.

Due to availability of impedance-pH-metry (see above), which enables a more comprehensive diagnosis, this modality is becoming less important.

In case of severe peristalsis disorders (e.g., follow-up of operated esophageal atresia), GER is difficult to detect; and therefore, pH-monitoring represents a better, more economic choice.

### Gastroesophageal reflux: imaging

GER detection is possible using almost all imaging modalities, with varying sensitivity and specificity.

#### Sonography

**Fig. 3:**
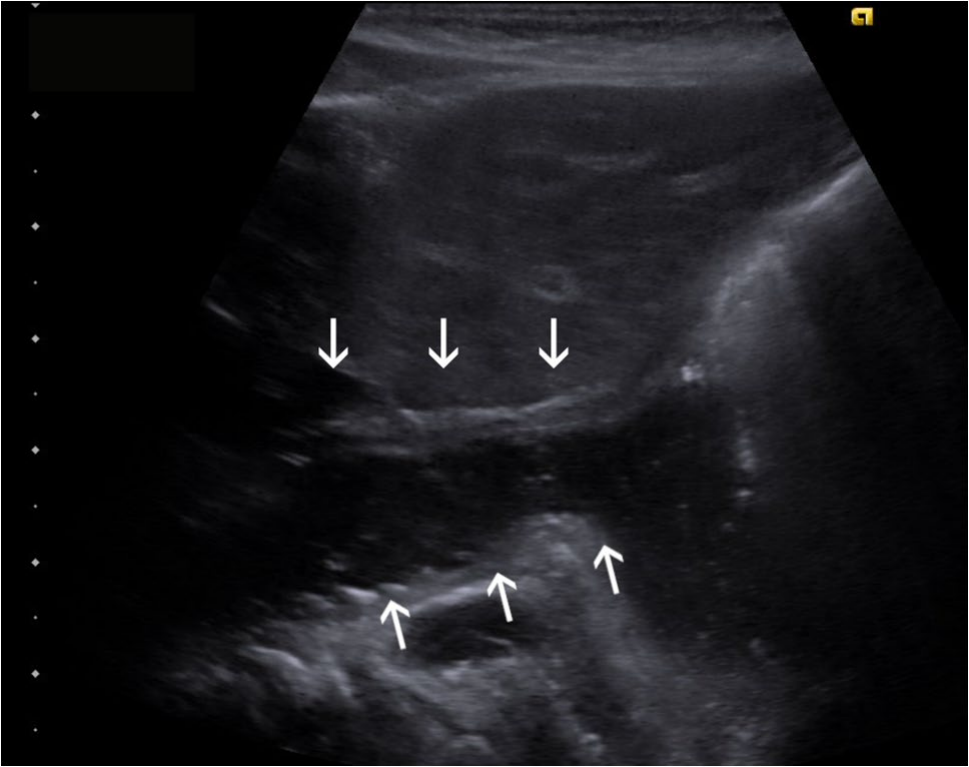
A left oblique parasagittal ultrasound image of the gastroesophageal junction in a 3-month-old boy. The cardia and distal esophagus are wide open (*arrows*) and there is reflux into the distal esophagus

Ultrasound enables real-time GER detection by imaging the gastroesophageal junction in a parasagittal plane (Fig. 3).

The following quantitative parameters can be assessed: (a) length of abdominal esophagus; (b) esophageal wall thickness; (c) esophageal diameters in short axis, and (d) rough estimation of the His angle [6]. Unfortunately, thus far, there is no grading available, especially for the differentiation between GER and GERD —which is essential for further appropriate patient management. Ultrasound can be used to detect causes of secondary GER, for example the gastric outlet should be evaluated for pyloric stenosis in small babies, as well as for gastric outlet obstruction (e.g., by an ectopic pancreas). The inverse position of the mesenteric vessels (superior mesentery vein to the left of the superior mesenteric artery) suggests malrotation [7].

#### Conventional radiographs

An air-filled esophagus detected on chest radiographs should raise the suspicion of GER, especially in a child with recurrent respiratory infections. A pneumoesophagus is frequently detected on chest radiographs in patients receiving supplemental oxygen by facial mask. It should be noted that nasogastric tubes provoke GER by disturbing the closure of the lower esophageal sphincter.

### Upper gastrointestinal series (barium swallow)

There are no published standards on how to perform an upper gastrointestinal seris (UGIs) and which/how GER provoking tests should be conducted, in fact over the past two decades, there have been almost no publications on the topic in general. A PUBMED search with keywords “Gastroesophageal Reflux[Mesh] AND Fluoroscopy[Mesh]” and applying an age filter (“birth-18 years”) retrieved no relevant studies; the one related paper (published in 2008) evaluated feeding strategies in neurologically impaired patients suffering from GER [8]. Carruci and Levine published guidelines for adult patients emphasizing the high sensitivity of double-contrast barium studies, especially for detection of esophagitis [9, 10]. Others reported GER detection with low sensitivity and specificity [11]. Aksglaede calculated a specificity of 100% and a sensitivity of 52% for UGI (compared to 24-h pH monitoring) [12]. For UGIs, DeBoer et al. published a sensitivity of 47.3%, specificity 74.2%, and positive predictive value of 50%, but there was no information given on either technique or provocative maneuver use (in particular, whether a water-siphon test was performed) [13]. Thompson et al. demonstrated in their study of 117 patients that UGIs detect unprovoked, spontaneous GER in only 26% of ph-monitoring–positive patients. Adding a water-siphon test to UGIs yielded a sensitivity of 70% at a specificity of 74% [14].

Since double-contrast barium studies are not feasible in most pediatric radiology units, the following UGIs technique has proven successful at the author’s institution:

A remote-operated fluoroscopy device is used. In order to keep radiation exposure as low as possible (ALARA principle), the device frame rate is set to 3 frames per second and most frequently, secondary capture images (Digital Imaging and Communications in Medicine screenshots) are used for documentation. Direct exposure (“a shot”) is only used for documentation of pathologies such as esophagitis.

Babies are positioned supine and older children are placed on a 45° tilted fluoroscopy table. Sometimes, young children do not want to swallow the contrast medium, and in these cases the following procedure can be helpful: a nasogastric tube is inserted and pushed forward to the gastric fundus. Under fluoroscopy control, the nasogastric tube is pulled back until its tip is located at the tracheal bifurcation level. A 50-ml contrast filled syringe is then connected and contrast medium injected using the bolus technique, where the first bolus is used to assess the esophageal motoric function, followed by anterior-posterior, lateral, and oblique (for gastroesophageal junction and HIS angle) projections of the contrast medium-filled esophagus. Care is taken to observe and document gastric emptying and correct position of the duodenal C, as well as of the duodenal–jejunal junction paramedian to the left, for exclusion of malrotation.

**Fig. 4:**
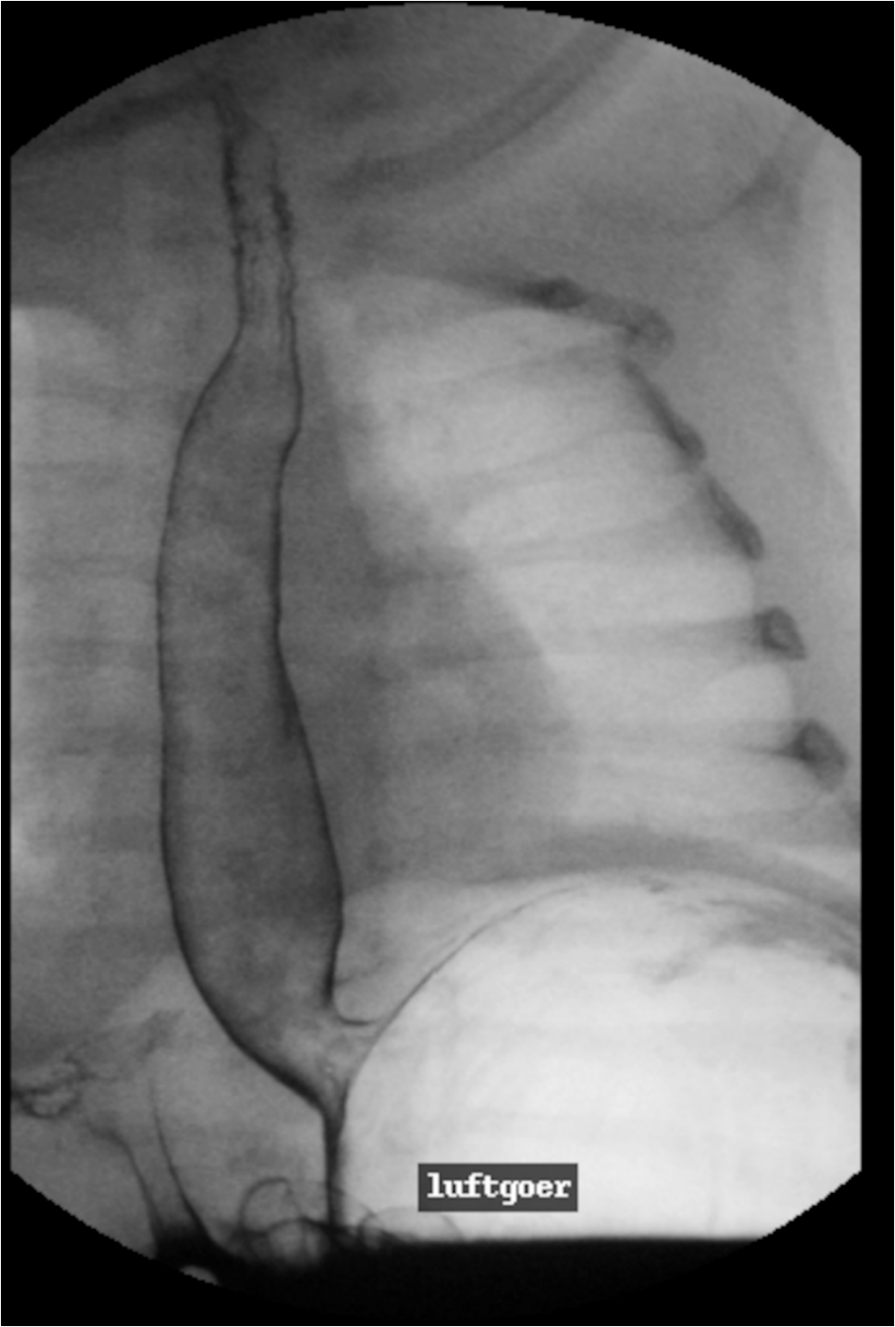
An erect, left anteroposterior secondary capture image from an upper gastrointestinal series in a 6-month-old girl whose impedance-pH-metry yielded pathologic results shows air reflux leading to a double-contrasted esophagus

**Fig. 5:**
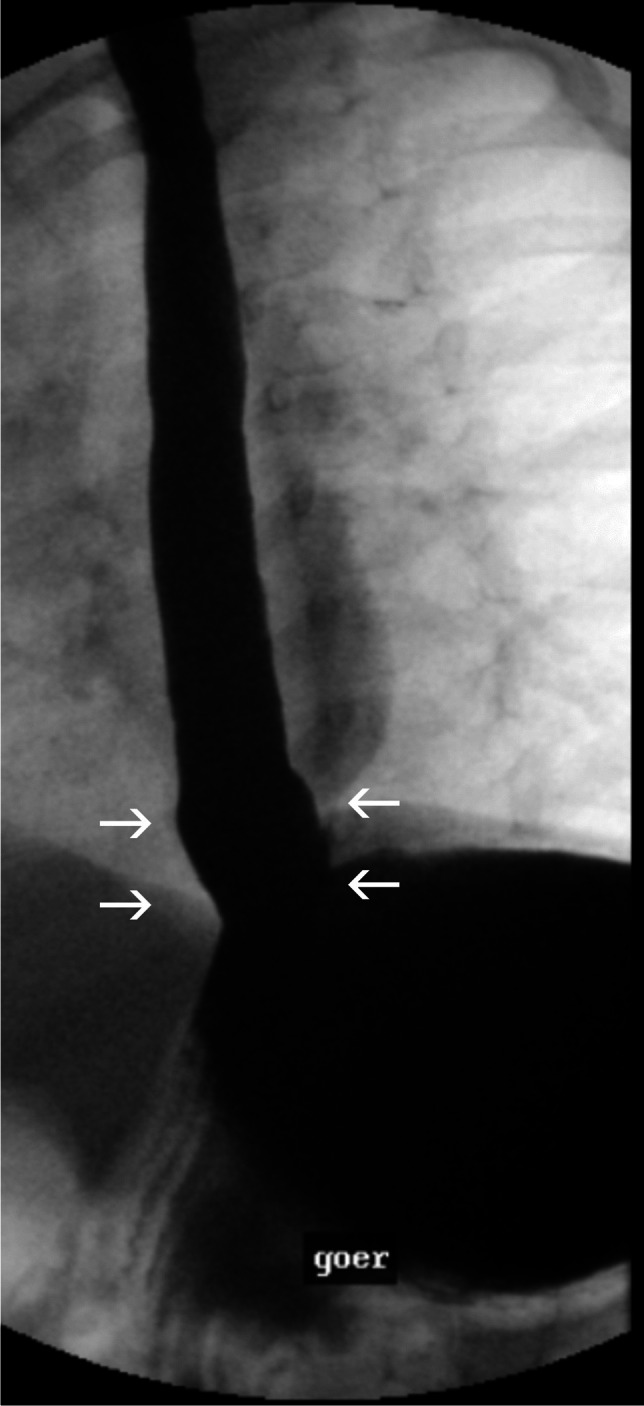
A left anterior oblique secondary capture image from an upper gastrointestinal series in a 3-year-old boy with pathologic findings on impedance-pH-metry. During the study, there was spontaneous gastroesophageal reflux and an axial hiatus hernia was identified (*arrows*), which was not seen during antegrade contrast medium passage, thereby confirming a sliding axial hiatus hernia

GER assessment is captured in three ways: (a) in the upright position—for air reflux (Fig. 4); (b) in the right lateral position—any occasion of spontaneous GER (Fig. 5), and (c) with the patient rotated to the left—for provocation using the water-siphon test, having pooled the contrast medium in the gastric fundus (by rotating the patient to the left). Patients swallow only a mouthful of water or tea (a maximum of three times) to provoke GER—any GER occurrence is of course documented. The UGIs ends with a secondary capture image of the upper abdomen, documenting gastric emptying and normal small bowel transit. If there is no gastric emptying by this time, the suspicion of a gastric motility disorder should be raised. GER is graded as shown in Table 2.

**Table 2:** Upper gastrointestinal series-based gastroesophageal reflux grading system—as used at the authors’ institution

	1 point	2 points	3 points
Spontaneous GER	Just immediately after water-siphon test	Anytime after water-siphon test	Before water-siphon test
Air reflux		Positive	
Water-siphon test	GER to tracheal bifurcation at any time	GER cranial to tracheal bifurcation at any time	GER at the first water-siphon test to cervical esophagus
Interpretation:	1-2 points	Minor GER	
	3-5 points	Moderate GER	
	6-8 points	Severe GER	

*GER* gastroesophageal reflux

We have analyzed results of manometry, standard pH-metry, endoscopy, and histology in 284 of our patients and found that for UGIs, including the water-siphon test, sensitivity is 86–90% at a specificity of 28–42% when compared to esophageal manometry and standard pH-metry (unpublished work). In patients with high-grade GER demonstrated by an UGIs, there were statistically significant higher rates of esophagitis at endoscopy and histology. As such, the UGIs is not the preferred screening tool due to its low specificity and use of ionizing radiation. Another critical issue is the type of contrast medium used for UGIs. Barium can be used in patients without swallowing disorders, whereas a water-soluble contrast medium is used for others. However, water-soluble contrast media hamper the detection of esophagitis.

At the author’s institution, the following workflow for GER evaluation was set up: Step 1: clinical examination, impedance-pH-metry, and an abdominal ultrasound in order to check gastrointestinal obstruction, e.g., pylorospasm in babies, or signs of malrotation in all age groups. Step 2: If impedance-pH-metry is pathologic, a patient is scheduled for an UGIs. Step 3: If the UGIs shows severe GER (6-8 points on our internal score), the patient has endoscopy with biopsy. Therefore, the reduced sensitivity of water-soluble contrast media for esophagitis is not an issue, because subsequent endoscopy and biopsy will detect it.

This simple approach allows smooth assessment of GER patients, is as non-invasive as possible, and enables initiation of appropriate therapy.

## Constipation - clinical and functional assessment

Functional constipation is a common problem in children, with a prevalence of 1–30%. Onset occurs in the first year of life and happens in 17–40%. Constipation is responsible for medical consultation in 25% of patients, and health costs of these children are three times higher than for non-constipated children. Furthermore, in 25% of these children, constipation persists into adulthood [15].

Risk factors for persistence of constipation are the following: (a) younger age at onset, (b) longer interval between onset of illness and first visit to the outpatient clinic, and (c) lower stool frequency at onset [14]. Early aggressive therapy (change in diet, exercise, appropriate fluid intake, laxatives if necessary) improves the outcome [16].

Constipation can be diagnosed using the Rome criteria, as depicted in Table 3 [3]. The presence of several symptoms should raise the suspicion of nonfunctional constipation (Table 4) and prompt for further diagnostics. [18].

**Table 3:** Rome criteria for diagnosing constipation [3]

Constipation is diagnosed by Rome IV criteria [17]	
≥2 criteria per month in children <4 years	2 or fewer bowel movements/week
	Excessive bowel retention
	Painful and hard stools
	Large-diameter stools
	Presence of large accumulations of stool in the rectum
Additional criteria for “toilet-trained” children	At least on episode of incontinence/week
	Large-diameter stool, which may obstruct the toilet

**Table 4:** “Warnings” for potential nonfunctional constipation [17]

Symptoms raising suspicion of non-functional constipation (“Warnings”)
Constipation starting in the 1^st^ month of life
Delayed passage of meconium beyond 48 h
Failure to thrive
Abdominal distension with explosive stools and/or intermittent diarrhea
Empty rectum
Tight anal sphincter and manual exploration
Sacral dimple with hairs
Lumbosacral pigment disorder
Neurological abnormalities
Blood deposits without fissures
Explosive stool on rectal examination
No history of retention and encopresis
Refractory constipation

The following examinations are available:

*High-resolution anorectal manometry *is an examination method for anorectal function diagnostics based on pressure measurements [18]. Functional tests of the anal sphincter apparatus are carried out using an anorectal pressure-measuring catheter (electronic or water-perfused) with an inflatable rubber balloon fixed to the tip of the catheter. The measuring points of the perfusion channels are attached spirally at defined intervals [18].

**Fig. 6:**
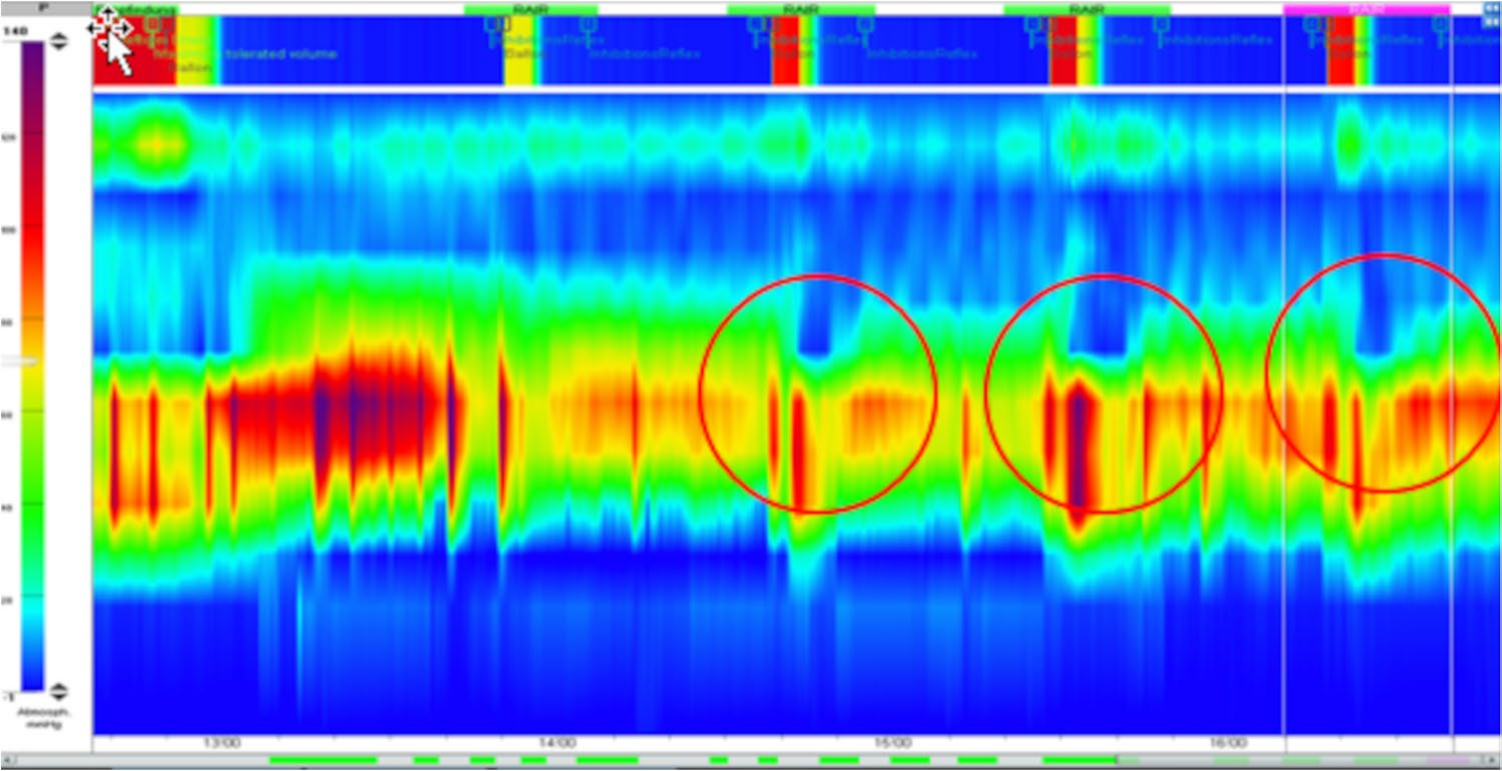
A screenshot from a high-resolution anorectal manometry study with color-coded intraluminal pressure tracings in a 6-year-old girl with constipation. Note the normal rectoanal inhibition reflex (*circles*)

The measuring catheter is inserted into the anorectum, and the balloon must be placed rectally 3–5 cm above the anal canal. The examination is carried out in the left lateral decubitus position and according to the standardized examination protocol of the International Anorectal Physiology Working Group [19]. The individual pressure values and pressure changes are color-coded (Fig. 6) [18].

High-resolution anorectal manometry is used for the assessment of defecation disorders, constipation, and fecal incontinence. It allows the determination of the resting and pinching pressures, the threshold values for perception, and the urge to defecate. The function of the sphincter apparatus can be assessed by standardized test maneuvers, such as coughing or the balloon expulsion test.

**Fig. 7:**
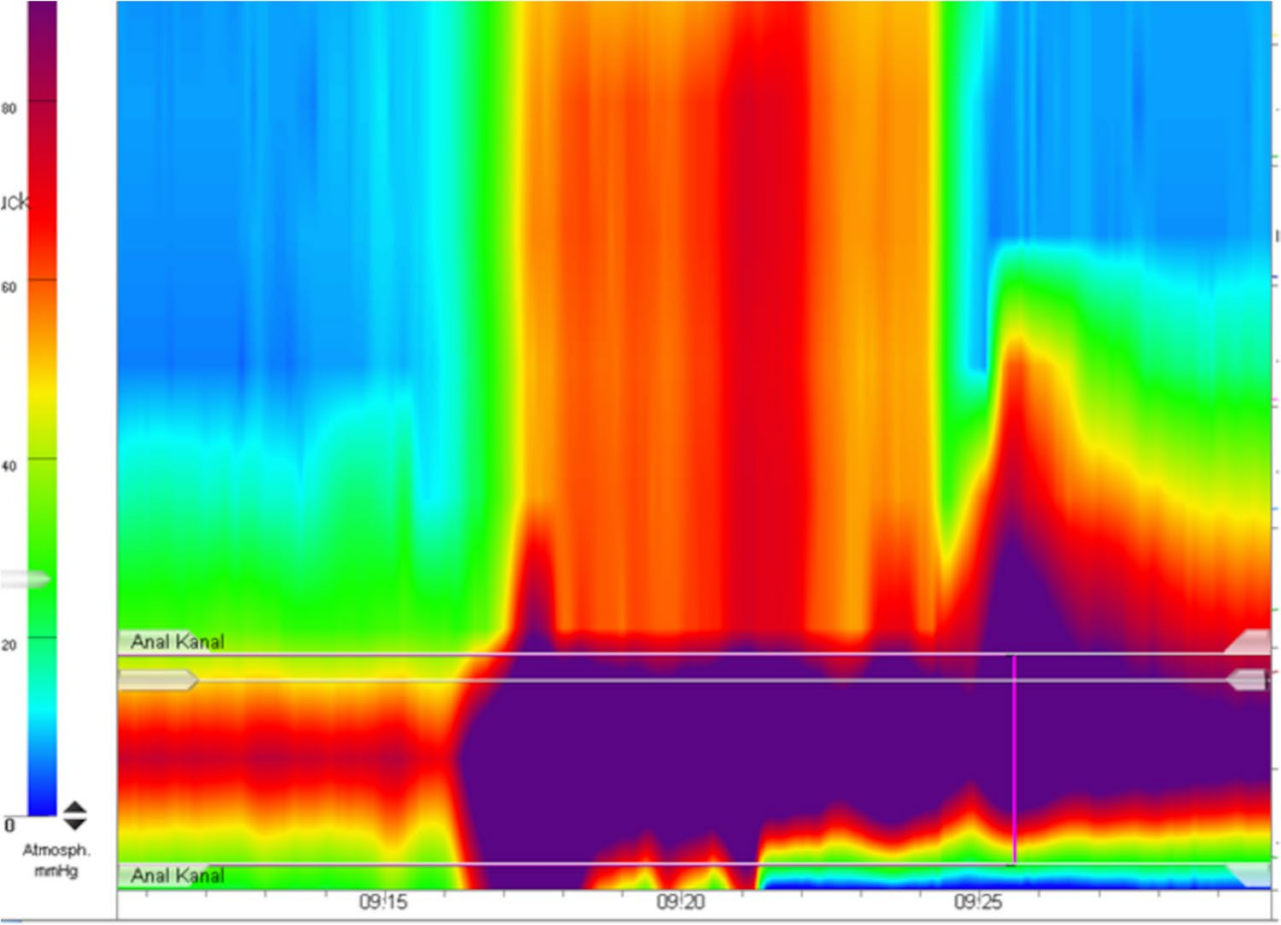
A screenshot from a high-resolution anorectal manometry study with color-coded intraluminal pressure tracings in a 4-year-old boy. There a is no rectoanal inhibition reflex detectable and the sphincter tone is significantly increased, indicating Hirschsprung’s Disease

The reliable, reproducible proof of the rectoanal inhibition reflex (RAIR—reflectory pressure drop of the inner anal sphincter after balloon inflation) allows the exclusion of Hirschsprung’s disease (Figs. 6 and 7). Care must be taken, as “slow waves” and “ultra-slow waves” of the sphincter can mimic a RAIR. Moreover, in patients with a significantly dilated rectum, the diameter of the inflated balloon catheter is smaller than that of the rectal catheter and therefore, RAIR cannot be induced. In such cases, therapy for ensuring stool evacuation should be initiated and high-resolution anorectal manometry subsequently repeated.

No clearly reproducible RAIR prompts further diagnostics and imaging, completed by rectal suction or full-thickness biopsy. The diagnosis of Hirschsprung’s disease can only be made by the gold standard— that being positive histology.

### Constipation - imaging

**Fig. 8:**
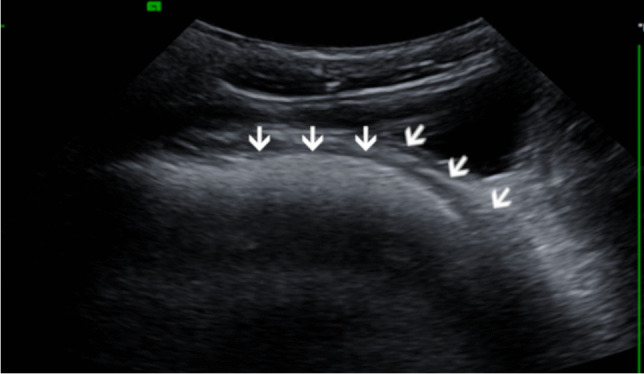
A transverse ultrasound scan of the pelvis in a 10-year-old boy with a clinical diagnosis of constipation shows a huge coprolite (*arrows*)

*Sonography* allows the entire bowel to be examined except loops hidden by gas, although the ascending and descending colon can be scanned even in these cases. Ultrasound can depict colon wall hypertrophy in stercoral colitis, as well inflammatory changes and coprolites (Fig. 8) [20].

**Fig. 9:**
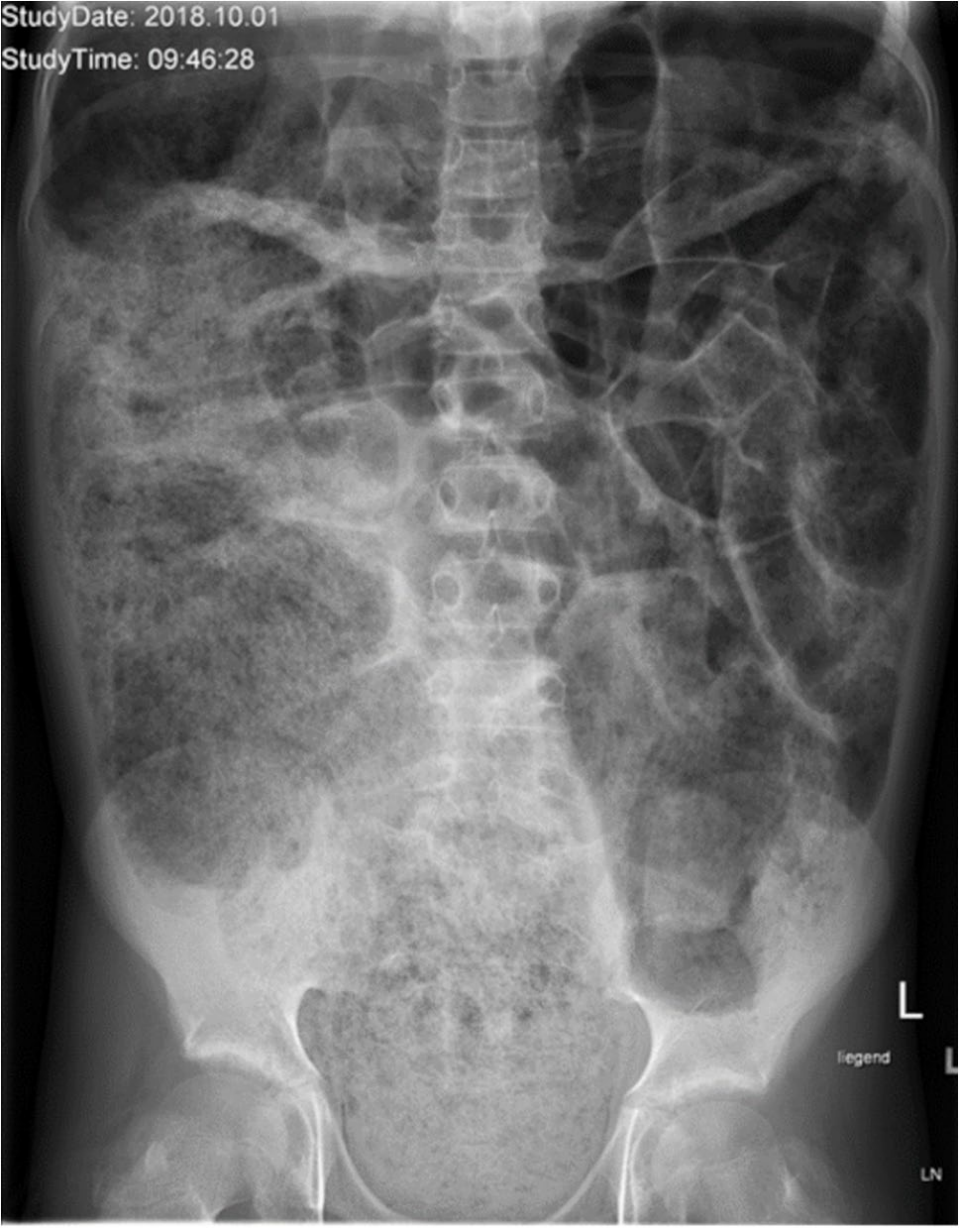
An anteroposterior abdominal radiograph in a 10-year-old boy with constipation (the same patient as in Fig. 8) shows massive fecal overloading and small bowel dilatation. The Barr score is 21 points—well above the normal limit of 10 points

*Conventional radiographs* can help to determine which parts of the gastrointestinal tract are dilated, demonstrate the amount of stool impaction (Fig. 9), show bezoars, and assess rectal diameter, which should normally be more than one-third and less than one-half of the pelvic diameter. An elongated sigmoid colon can be detected in all constipated children except neonates. Barr et al. published a scoring system for semi-quantitative assessment of fecal retention [21].

**Fig. 10:**
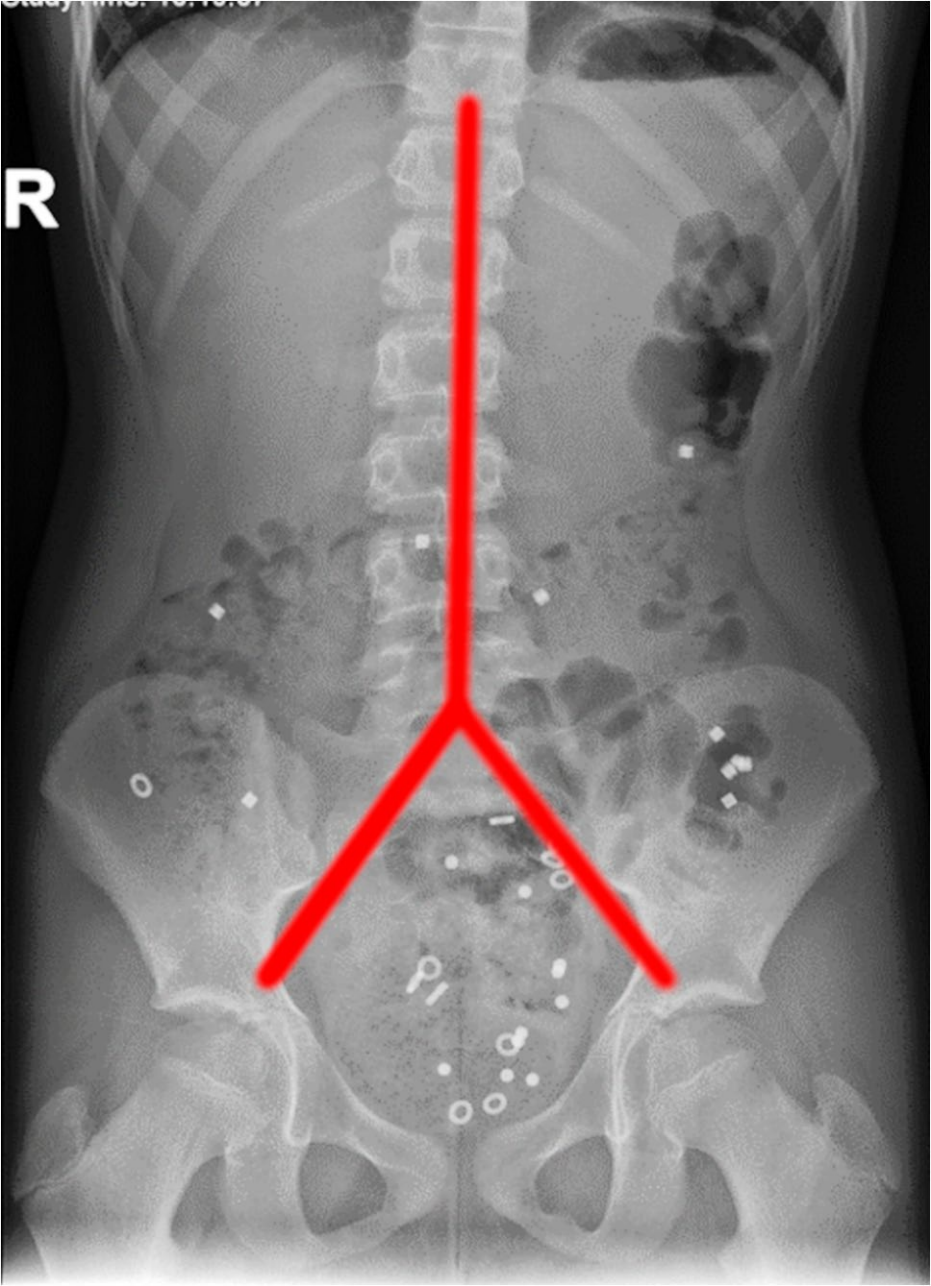
An anteroposterior abdominal radiograph in an 8-year-old girl with constipation referred for colon transit time (CTT) estimation. Markers were given for six days and the radiograph taken on day 7. For evaluation, the abdominal area is divided into three parts (*red lines*). There are four markers in the right quadrant, seven in the left quadrant, and 19 in the pelvic area. The transit times can be calculated by multiplying the markers by a factor of 2.4. Therefore, the transit time fare 9.6 h, 16.8 h, and 45.6 h for the right hemi-colon, left hemi-colon and recto-sigmoid, respectively, resulting in a total CCT of 72 h. Values for the right and left hemi-colon are just above the normal limits, while the time for the recto-sigmoid colon and total CTT are prolonged. The final diagnosis was functional constipation

*Colon transit time *is a method whereby patients swallow radiopaque markers and their GI transit is monitored on abdominal radiographs. Markers can be given all at once or on several days, sequentially [22]. Vande Velde reported a modification of the technique for children [23]. Polythene radiopaque markers are swallowed on six consecutive days, followed by an abdominal radiograph on day 7. Based on marker number and distribution, the colon transit time can then be calculated (Fig. 10). Depending on the technique used, normal colon transit time values for children and adolescents are reported to be a median of 38.8 h, with an upper limit of 80.0 h [23]. Colon transit times may also be assessed using scintigraphy [24].

*Defecography* represents the gold standard for further evaluation in cases of pathologic high-resolution anorectal manometry. Fotter et al. have described the procedure in detail [25]. Patient preparation consists of rectal cleaning, with a small enema the evening before, and another 2 h before the examination. By placing a rectal catheter, the rectum, sigmoid, and descending colon are filled with barium suspension from the anus to the left colonic flexure. Documentation is by intermittent fluoroscopy.

**Fig. 11:**
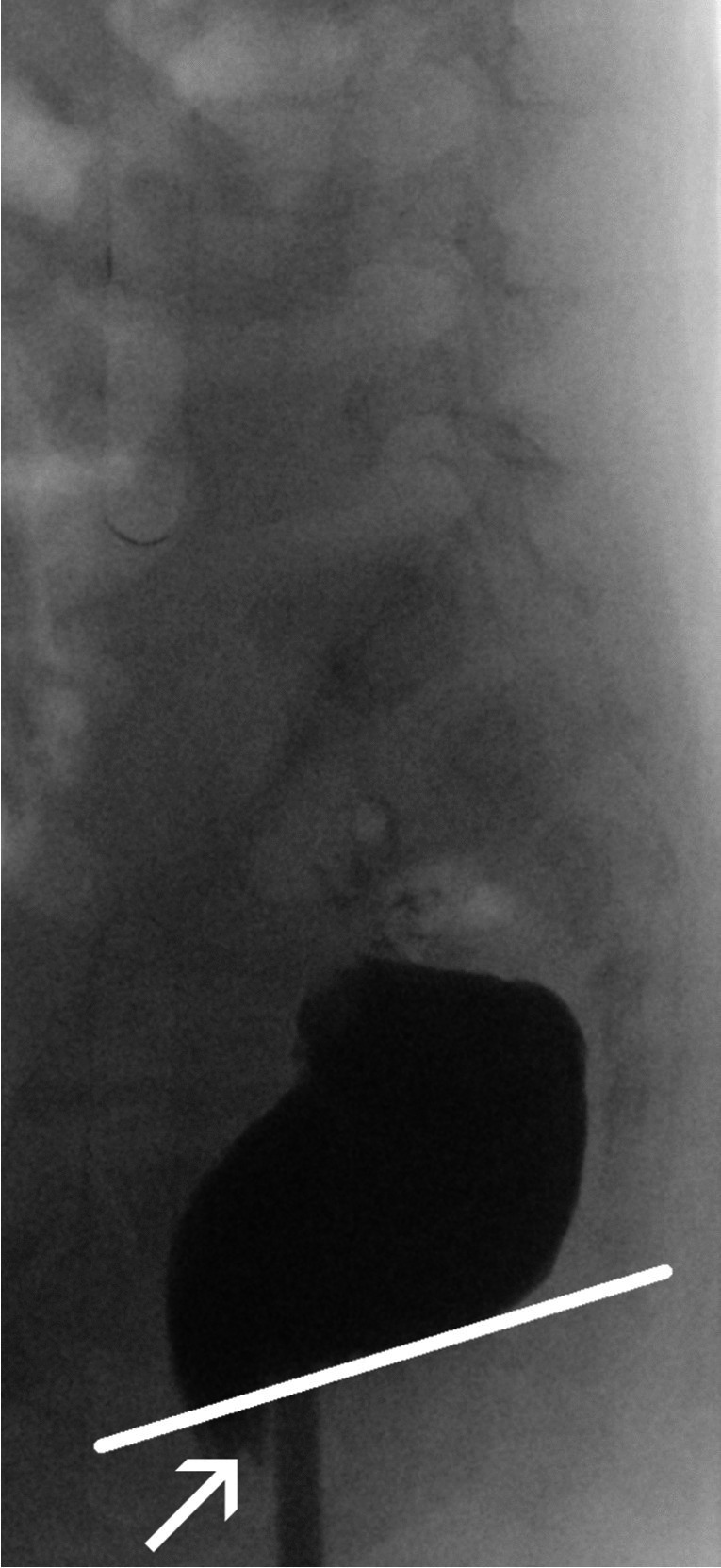
A screenshot lateral radiographic image of the rectum obtained during defecography in a 4-year-old girl diagnosed with functional constipation. The rectoanal inhibitory reflex (RAIR) is assessed by drawing a tangent to the caudal contour of the rectum (*line*) and (in the case of a positive RAIR) noting a bulge (*arrow*) below this tangent

**Fig. 12:**
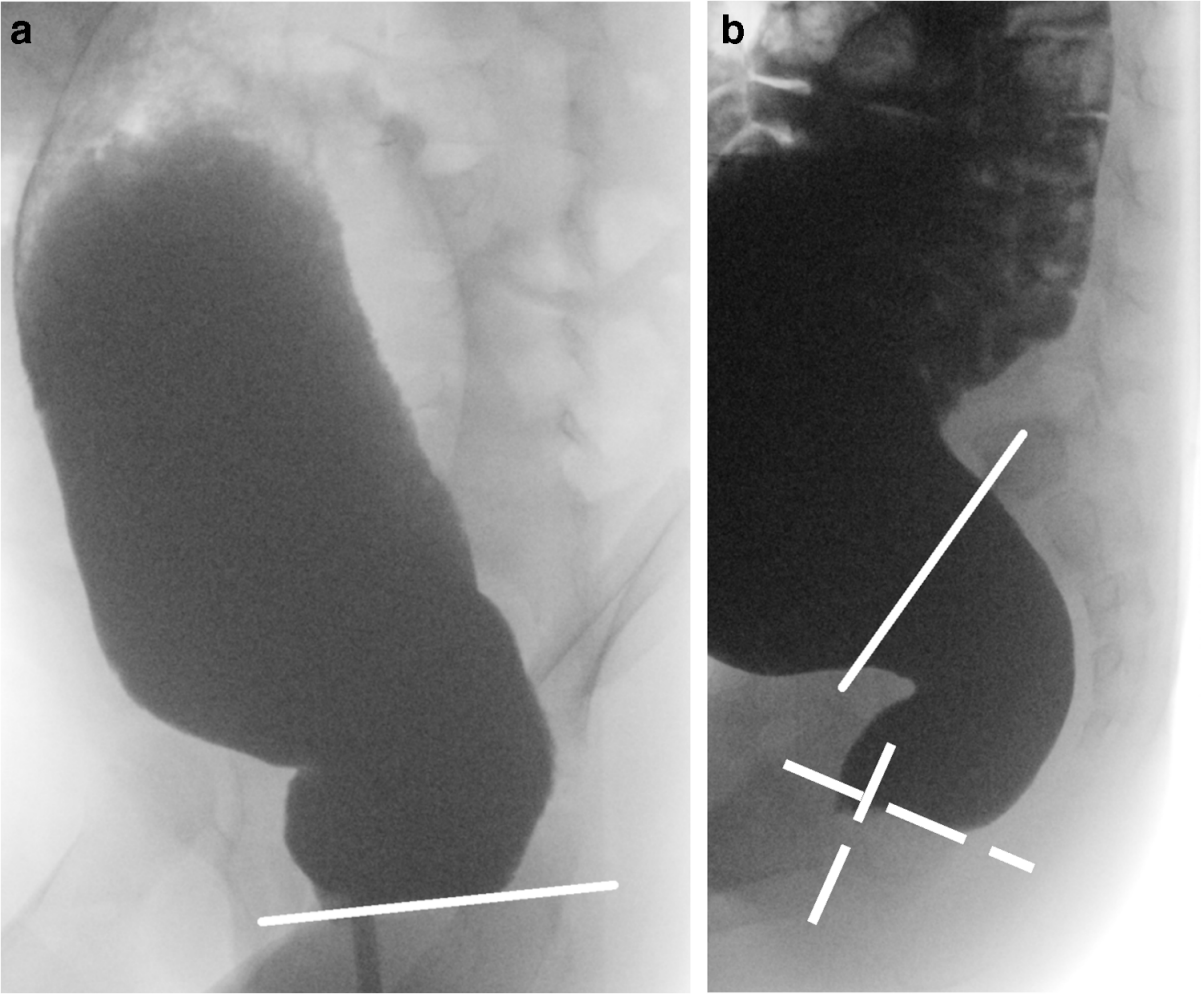
Lateral radiographs of the rectum obtained during a defecography performed to determine the transition zone in a 3.5-year-old girl with Hirschsprung disease. **a** There is no rectoanal inhibitory reflex detectable since there is no bulge below the tangent (*line*) on the caudal rectal contour. **b** During active defecation, there is no opening of the anal channel and no reduction in the anorectal angle (*broken* *lines*). Note the transition zone (*solid* *line*)

RAIR can also be detected on defecography (Fig. 11), and an absent RAIR points to Hirschsprung’s disease—where the transition zone can be defined as the interface between the aganglionic segment and the dilated more proximal normal colon (Fig. 12).

The catheter is subsequently removed and the patient is asked to actively defecate. At this point in time, pelvic floor relaxation with flattening and opening of the anorectal channel can be observed and pathologies such as rectoceles and intussusceptions can be detected. The procedure ends after requesting the patient to close their sphincter (for continence assessment, demonstrated by an anorectal angle of 90 degrees or less).

Since it is a functional investigation and dynamics are slow, a frame rate of 1-2 frames/s can be used and dose settings reduced, as well as an exposure curve for high contrast, selected on the fluoroscope device.

Within the diagnostic odyssey, defecography represents the final examination before biopsy in patients with therapy-refractory constipation. High-resolution anorectal manometry yields pathologic results and therefore the suspicion of Hirschsprung’s disease is raised.

In conclusion, GER and constipation are frequent reasons to seek medical support. Using a staged approach of available diagnostic procedures helps to be as non-invasive as possible, adheres to the ALARA principle and represents “patient-tailored medicine.” Finally, it must be considered that up-to-date diagnostic equipment is not available everywhere, whereas fluoroscopy units are widespread. Using appropriate technique, fluoroscopy can contribute to the diagnostic pathway.

## Data Availability

Data sharing is not applicable to this article as no datasets were generated or analyzed during the current study. Only example images are included.
